# The expression and survival significance of sodium glucose transporters in pancreatic cancer

**DOI:** 10.1186/s12885-021-09060-4

**Published:** 2022-01-28

**Authors:** Jiali Du, Jichun Gu, Junyuan Deng, Lei Kong, Yujie Guo, Chen Jin, Yun Bao, Deliang Fu, Ji Li

**Affiliations:** 1 Department of Pancreatic Surgery, Huashan Hospital, Fudan University, 12 Middle Wulumuqi Rd, Shanghai, 200040 People’s Republic of China; 2 Department of Pathology, Huashan Hospital, Fudan University, 12 Middle Wulumuqi Rd, Shanghai, 200040 People’s Republic of China

## Abstract

**Background:**

Sodium glucose transporters (SGLTs) play vital roles in glucose uptake in many solid cancers, including pancreatic cancer (PC). However, their expression profile in pancreatic cancer and correlation with prognosis are not clear. Thus, we aimed to analyse the expression profile and prognostic significance of SGLT-1 and SGLT-2 in PC.

**Methods:**

Eighty-eight patients with pancreatic ductal adenocarcinoma (PDAC) undergoing surgery in Huashan Hospital, Fudan University, from July 2017 to June 2020 were enrolled in the study. Specimens for immunohistochemistry were obtained through surgical resection. Bioinformatics analysis was performed based on the Gene Expression Omnibus (GEO), Oncomine and The Cancer Genome Atlas (TCGA) databases. The statistics were calculated using IBM SPSS Statistics, version 20 and R 4.1.1. *P* values lower than 0.05 were considered to indicate statistical significance.

**Results:**

SGLT-1 but not SGLT-2 was significantly overexpressed in PDAC. Survival analysis showed that the median overall survival (OS) and progression-free survival (PFS) of patients with high SGLT-1 expression were significantly longer than that of patients with low SGLT-1 expression. Cox regression indicated that high SGLT-1 expression was an independent predictor for a better prognosis, while residual tumour status (R1 and R2) was an independent risk factor for a poor prognosis. Finally, PDZK1-interacting protein 1 (PDZK1IP1), a protein participating in the generation of reactive oxygen species, was overexpressed in PDAC and its expression was significantly correlated with SGLT-1.

**Conclusions:**

SGLT-1 but not SGLT-2 was overexpressed in PDAC, and the overexpression of SGLT-1 could be a predictor of a better prognosis. Residual tumour status (R1 and R2) was a risk factor for poor prognosis and disease progression.

**Supplementary Information:**

The online version contains supplementary material available at 10.1186/s12885-021-09060-4.

## Introduction

Pancreatic ductal adenocarcinoma (PDAC), accounting for 90% of pancreatic cancer (PC) cases, is the seventh leading cause of cancer deaths [1, 2]. Due to the absence of symptoms and significant biomarkers used for early screening, most patients lose the opportunity for radical surgical resection. Thus, it is crucial to identify novel and valid biomarkers for the diagnosis and treatment of PC.

The increased uptake and metabolism of glucose is an important hallmark of cancer [3–5]. PDAC is a remarkably stroma-rich, vascular-poor, hypoperfused tumour, leading to deficient drug delivery, which is the main cause of drug resistance. Interestingly, glucose delivery to tumour cells do not seem to be damaged [6]. Thus, it is possible to explore novel targets in the process of glucose uptake and metabolism in PDAC.

Sodium-dependent glucose transporters (SGLTs) are active transporters or symporters encoded by the SLC5 gene family, and the energy of transportation is provided by the sodium gradient across the cell membrane. It was found that inhibiting SGLT-2 could block glucose uptake and suppress tumour proliferation and invasion in a xenograft model of pancreatic cancer [7, 8]. It is worth noting that SGLT-2 was predominantly expressed in lung premalignancy and early-stage, well-differentiated lung adenocarcinoma (LADC), and as lung tumours progress to advanced and poorly differentiated cancers, they upregulate glucose transporter 1 (GLUT-1) as the dominant transporter [9]. However, the expression level of SGLTs in PC patient samples and its correlation with the clinical outcome of PC are not clear, and the connection between the expression of GLUT-1 and SGLTs in PC have not been studied.

Additionally, SGLT-1 has been reported to be correlated with MAP17-induced ROS (reactive oxygen species) production in cancer cells, and the inhibition of this membrane transporter inhibits MAP17-dependent ROS increase and the proliferation of tumour cells [10–12]. MAP17 is a small, nonglycosylated, membrane-associated 17-kDa protein that acts as an atypical anchoring site for PDZK1 and interacts with the NaPi-IIa/PDZK1 protein complex in renal proximal tubular cells, so it is also called PDZK1-interacting protein 1 (PDZK1IP1) [11, 13]. Notably, it could stimulate the specific Na-dependent transportation of mannose and glucose in Xenopus oocytes and human tumour cells and was overexpressed in a variety of human carcinomas, enhancing the tumorigenic phenotype by increasing intracellular ROS [12, 14–19]. ROS are oxygen-derived molecules, mostly the free radicals superoxide anion O_2_^−^ and hydroxyl radical -OH, and they can promote cancer development, chemoresistance, and relapse by causing oxidative DNA damage and genomic instability and modifying gene expression [20–23]. It has been reported that the levels of ROS are notably increased in patients with pancreatic cancer and are involved in the progression, drug resistance, recurrence and metastasis of pancreatic cancer [24, 25]. However, excessive concentrations of ROS can lead to the induction of cell cycle arrest and cell death, consistent with reports that higher expression of MAP17 was correlated with better prognosis in laryngeal and cervical cancers [10, 11, 25, 26].

To study the mechanisms of glucose ingestion by PC tumour cells and explore novel effective targets for PC diagnosis and treatment, we examined the expression profile and survival relevance of SGLTs in PC and elucidated the specific prognostic significance of SGLT-1, in contrast to GLUT-1, which has been reported to be correlated with a poor prognosis. Given the specific expression pattern of SGLT-2 in LADC and the unique role of SGLT-1 in the generation of ROS stated above, we then explored the mechanism underlying the specific survival significance of SGLT-1 from these two aspects. We identified the expression profiles of SGLT-1 and SGLT-2 and suggested that SGLT-1 could be a biomarker for the diagnosis, treatment, and prognosis of PC. Additionally, we hypothesized that SGLT-1 might play an important role in ROS generation in addition to glucose uptake in PC.

## Materials and methods

### Patient and tumour samples

Following the ethics approval of the Institutional Review Board of Fudan University Huashan Hospital (Shanghai, China), the paraffin-embedded surgical specimens and data of 88 patients who underwent radical surgery for PDAC from July 2017 to June 2020 were retrospectively collected from the Department of Pancreatic Surgery of Huashan Hospital, Fudan University. Before surgery, written consent for the inclusion of related material was retrieved from all the patients in this study. Any information that could identify the patients was not included in this article. All human tissues, including 88 tumour tissues and 50 adjacent normal tissues, were intraoperatively removed and obtained from the Department of Pathology in the form of consecutive resection slices. The inclusion criteria were as follows: (i) patients were pathologically diagnosed with PDAC after radical surgery, and (ii) the medical history of the patients was complete. The exclusion criteria were as follows: (i) patients underwent palliative surgery but not radical surgery for PDAC, and (ii) the final pathological diagnosis was not PDAC.

### Immunohistochemistry (IHC)

The samples were subjected to antigen retrieval by incubation in EDTA antigen retrieval buffer (pH 9.0) for 15 min at 100 °C. Endogenous peroxidase was blocked by incubation with 3% H_2_O_2_ for 25 min, followed by washing in PBS 3 times for 5 min each time. The cells were incubated with 3% bovine serum albumin (BSA) to block other antigens. Incubation with primary antibodies (Abcam ab15309 (1:200), Abcam ab14685 (1:200), Novus Biologicals NBP1–92384 (1:250) and Abcam ab85626 (1:250)) was performed overnight at 4 °C. After washing 3 times, the samples were incubated with secondary antibody labelled with HRP (GB23303, 1:200, Servicebio) for 50 min at room temperature, followed by washing and incubation with diaminobenzidine substrate (DAKO) for a controlled period under a microscope. For each experiment, human kidney was used as a positive control, and overnight preincubation of the antibodies with the respective competitor peptides was used as a negative control. Counterstaining was performed with diluted Harris haematoxylin (KIGENE). All microscopic slides were scanned with a 3DHISTECH CaseViewer system. The antibody information of ab15309: rabbit polyclonal to glucose transporter GLUT-1, Abcam, synthetic peptide within human GLUT-1 aa 450 to the C-terminus (C-terminal) was previously validated [27]. The antibody information of ab14685: rabbit polyclonal to SGLT-1, Abcam, synthetic peptide corresponding to amino acids 603–623 of human SGLT-1 was previously validated [28]. The antibody information of NBP1–92384: rabbit polyclonal to SGLT-2, Novus Biologicals, developed against recombinant protein corresponding to amino acids FHEVGGYSGLFDKYLGAATSLTVSEDPAVGNISSFCYRPRPDSYHLL was previously validated [9].

### Image analysis

Two pathologists scored all immunohistochemical staining of SGLT-1, SGLT-2 and GLUT-1 for the intensity of staining and percentage of positively stained tumour cells independently under a high-power field (HPF), with a final magnification of 10 × 40. The staining intensities of PDAC tumour cells were scored in 4 degrees: 0 (no staining), 1 (low), 2 (intermediate), and 3 (high). The percentage of tumour cells staining positive for each degree in each tissue slide was scored in 5 levels: 0 (0%), 1 (1–20%), 2 (21–40%), 3 (41–60%), 4 (61–80%), and 5 (81–100%). The scores for each tissue slide were calculated as follows: 1 * percentage score of tumour cells staining low + 2 * percentage score of tumour cells staining intermediate + 3 * percentage score of tumour cells staining high.

The final expression score is the average of the interpretation results of two pathologists. The patients were dichotomized into a low expression group and a high expression group according to the staining intensity of different proteins.

### Bioinformatics analysis

All three expression microarray series, GSE15471, GSE28735 and GSE62165, containing PDAC tumour (*n* > 30) and nontumour samples in the Gene Expression Omnibus (GEO) database (https://www.ncbi.nlm.nih.gov/geo/) were downloaded (date of access for databases: 2020–10–28) [29–31]. The necessary details of the GEO series are summarized in Table 2. The mRNA expression of SGLT-1 (SLC5A1) and SGLT-2 (SLC5A2) in pancreatic cancer was examined again in all datasets containing human PDAC tumour (*n* > 30) and nontumour samples in the Oncomine database (https://www.oncomine.org/), a cancer microarray database and web-based data-mining platform, and one of the two datasets was the same as GSE15471 in GEO, contributed by the same authors (date of access for databases: 2021–03–02) [29, 32]. The Gene Expression Profiling Interactive Analysis (GEPIA) database (http://gepia.cancer-pku.cn/) was used to perform Kaplan–Meier survival analysis and Cox regression based on the mRNA expression level of glucose transporters in The Cancer Genome Atlas (TCGA) database [33]. In data mining of TCGA Database, mRNA-seq of primary PAAD (pancreatic adenocarcinoma) tissues was retrieved from the TCGA-PAAD cohort, and clinic data of TCGA-PAAD cohort was downloaded from UCSC Xena (http://xena.ucsc.edu), an online exploration tool for public and private, multi-omic and clinical/phenotype data. Among them, 172 patients had both mRNA-sep data and clinical data. After excluding 1 sample without the complete specific TNM stage data, 1 performed with neoadjuvant therapy and 26 samples with histological type other than PDAC, 144 patients having both mRNA-seq-FPKM-UQ data and clinic data were finally enrolled in this study. The integration of data was carried out in R 4.1.1. Survival analysis between groups with different SGLT-1 (SLC5A1) expression and univariate/multivariate analysis of predictive factors for overall survival (OS) and progression-free survival (PFS) were conducted by packages pacman, tidyverse, survival, survminer and plyr of R. Factors with *P <* 0.1 in univariate analysis would be included in multivariate analysis then. And *P <* 0.05 was defined as the threshold.

### Statistical analysis

The expression levels of glucose transporters in tumour and adjacent normal tissues were compared by Student’s *t*-test or Wilcoxon analysis according to whether the scores in each group conformed to a normal distribution. The correlation of the expression level of different proteins was calculated via Pearson correlation. Differences between different categorical variables were assessed using the chi square test or Fisher’s exact test. Survival curves were plotted using the Kaplan–Meier method and analysed using the log-rank test. OS was calculated as the time (days) between surgery and death from any cause. The multivariate Cox proportional hazards model was used to estimate the adjusted HR and to determine independent factors associated with survival using significant factors from the univariate analysis and other clinically meaningful factors as covariates. The hazard ratio (HR) and its corresponding 95% confidence interval (CI) of factors excluded from the Cox proportional hazards model equation were calculated with the all-entering method in Cox regression. All *P* values lower than 0.05 were considered to indicate statistical significance. The statistics above were calculated using IBM SPSS Statistics, version 20. All statistical graphs were drawn with GraphPad Prism for Windows, version 8.0.2 and processed by Adobe Illustrator CC 2018.

## Results

### Expression of SGLT-1 and SGLT-2 in PDAC

**Fig. 1:**
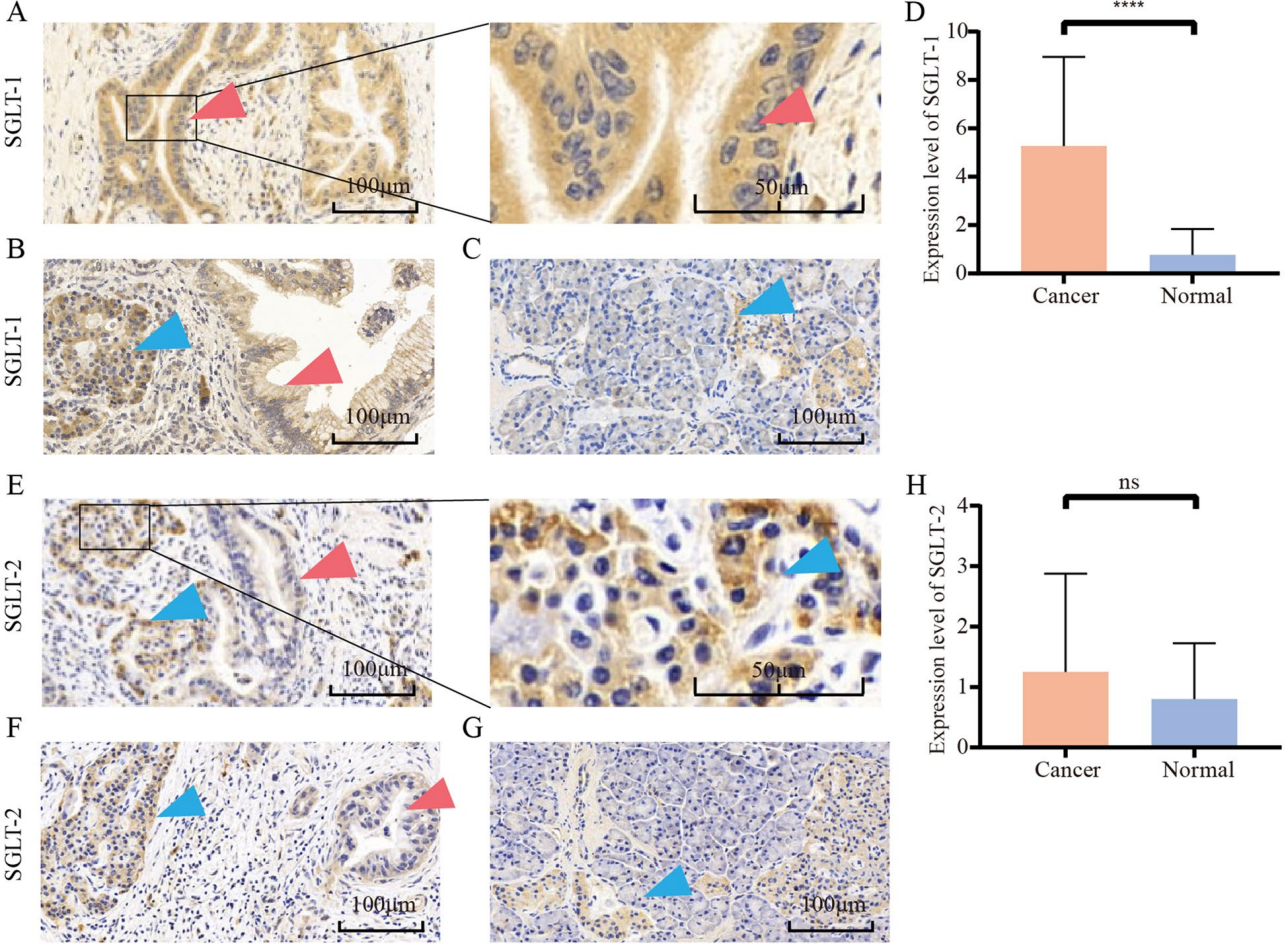
IHC analysis of the expression of SGLT-1 and SGLT-2 in PDAC tumour tissue and adjacent normal tissue. **A** SGLT-1 was predominantly overexpressed in the cytoplasm of tumour cells. **B** SGLT-1 was also overexpressed on the membrane of tumour cells. **C** SGLT-1 was positively expressed on islet cells but not in normal pancreatic cells. **D** The expression of SGLT-1 in tumour cells was significantly higher than that in normal pancreatic cells. **E** and **F** SGLT-2 was positively expressed in islet cells but not in pancreatic cancer. **G** SGLT-1 was positively expressed in islet cells but not in normal pancreatic cells. **H** Statistical comparison of the expression of SGLT-2 between pancreatic cancer and normal pancreatic ducts and acinar cells. (Red arrow, tumour cell; blue arrow, islet cells; ^****^ for *P*<0.0001; error bar: standard deviation; ns: not significant)

Among all 88 patients, 12 underwent preoperative chemotherapy or radiotherapy, and the remaining 76 underwent radical surgery directly. The baseline characteristics of the patients are shown in Table 1. IHC analysis showed that SGLT-1 was predominantly expressed in the cytoplasm and partly on the membrane of malignant cells (Fig. 1A and B). However, SGLT-2 staining was negative in most PDAC tumour samples, no matter which primary antibody for SGLT-2 was used. Only a few tumour cells in certain samples showed weak positive staining in the cytoplasm (Fig. 1E, F and Fig. S1). There was little antibody staining of SGLT-1 and SGLT-2 in normal tissue (Fig. 1C and G). In addition, both SGLT-1 and SGLT-2 had obviously positive staining on islet cells (Fig. 1B, C, F and G). SGLT-1 was significantly overexpressed in PDAC tumour cells (mean score: 5.273 vs. 0.760, *P* <  0.0001, Fig. 1D). But no significant difference was found in the expression level of SGLT-2 between tumour and adjacent normal tissue on serial slices with different primary antibodies. Mean IHC score of SGLT-2 in tumor and adjacent normal tissue was 1.250 vs. 0.800 when antibody of Novus Biologicals NBP1–92384 was used (*P* = 0.075, Fig. 1H), and was 1.284 vs 1.636 when antibody of Abcam ab85626 was used (*P* = 0.079, Fig. S1C).

**Table 1:** Correlation between expression of SGLT-1 and clinicopathological characteristics of patients

Features	No. of all patients (%)	SGLT-1 expression			No. of patients without preoperative chemotherapy or radiotherapy (%)	SGLT-1 expression		
		low	high	*P*		low	high	*P*
All	88 (100.0)	45	43		76 (100.0)	41	35	
Age (years)				0.396				0.246
< 64	43 (48.9)	20 (44.4)	23 (53.5)		36 (47.4)	17 (41.5)	19 (54.3)	
≥ 64	45 (51.1)	25 (55.6)	20 (46.5)		40 (52.6)	24 (58.5)	16 (45.7)	
Sex				0.767				0.802
Female	30 (34.1)	16 (35.6)	14 (32.6)		25 (32.9)	14 (34.1)	11 (31.4)	
Male	58 (65.9)	29 (64.4)	29 (67.4)		51 (67.1)	27 (65.9)	24 (68.6)	
Lymph node positive				0.817				0.807
< 1	40 (45.5)	19 (42.2)	21 (48.8)		36 (47.4)	18 (43.9)	18 (51.4)	
≤ 1 and < 4	31 (35.2)	17 (37.8)	14 (32.6)		26 (34.2)	15 (36.6)	11 (31.4)	
≥ 4	17 (19.3)	9 (20.0)	8 (18.6)		14 (18.4)	8 (19.5)	6 (17.2)	
Tumour size				0.538				0.506
≤ 4 cm	66 (75.0)	35 (77.8)	31(72.1)		57 (75.0)	32 (78.0)	25 (71.4)	
> 4 cm	22 (25.0)	10 (22.2)	12 (27.9)		19 (25.0)	9 (22.0)	10 (28.6)	
TNM stage				0.823				0.985
I–II	42 (47.8)	22 (48.9)	20 (46.5)		37 (48.7)	20 (48.8)	17 (48.6)	
III	46 (52.2)	23 (51.1)	23 (53.5)		39 (51.3)	21 (51.2)	18 (51.4)	
Grade				0.499				0.747
I	44 (50.0)	25 (55.6)	19 (44.2)		38 (50.0)	22 (53.7)	16 (45.7)	
II	26 (29.5)	11 (24.4)	15 (34.9)		21 (27.6)	10 (24.3)	11 (31.4)	
III	18 (20.5)	9 (20.0)	9 (20.9)		17 (22.4)	9 (22.0)	8 (22.9)	
SMA/SMV Invasion				0.964				0.897
No	53 (60.2)	27 (60.0)	26 (60.5)		45 (59.2)	24 (58.5)	21 (60.0)	
Yes	35 (39.8)	18 (40.0)	17 (39.5)		31 (40.8)	17 (41.5)	14 (40.0)	
History of diabetes				0.130				0.219
No	73 (83.0)	40 (88.9)	33 (76.7)		63 (82.9)	36 (87.8)	27 (77.1)	
Yes	15 (17.0)	5 (11.1)	10 (23.3)		13 (17.1)	5 (12.2)	8 (22.9)	

*SMV* Superior mesenteric vein, *SMA* Superior mesenteric artery

### Correlation between IHC expression of glucose transporters and clinicopathological characteristics

The clinical and pathological features of the patients without preoperative chemotherapy or radiotherapy are presented in Table 1. Because positive SGLT-2 expression was not found in PDAC samples, we investigated the association between the expression level of SGLT-1 and clinicopathological characteristics, including age (< 64 and ≥ 64 years old), sex (female and male), lymph node metastasis (< 1, ≤1 and < 4, and ≥ 4), tumour size (≤4 cm and > 4 cm), TNM stage (I–II and III), tumour pathological grade (I–II, III and IV), SMA/SMV invasion (yes and no), and diabetes history (yes and no). The patients were divided into two groups according to the expression level of SGLT-1. There was no significant difference in the expression of SGLT-1 for any of the features (Table 1).

### mRNA levels of SGLT-1 (SLC5A1) and SGLT-2 (SLC5A2) in PDAC

**Fig. 2:**
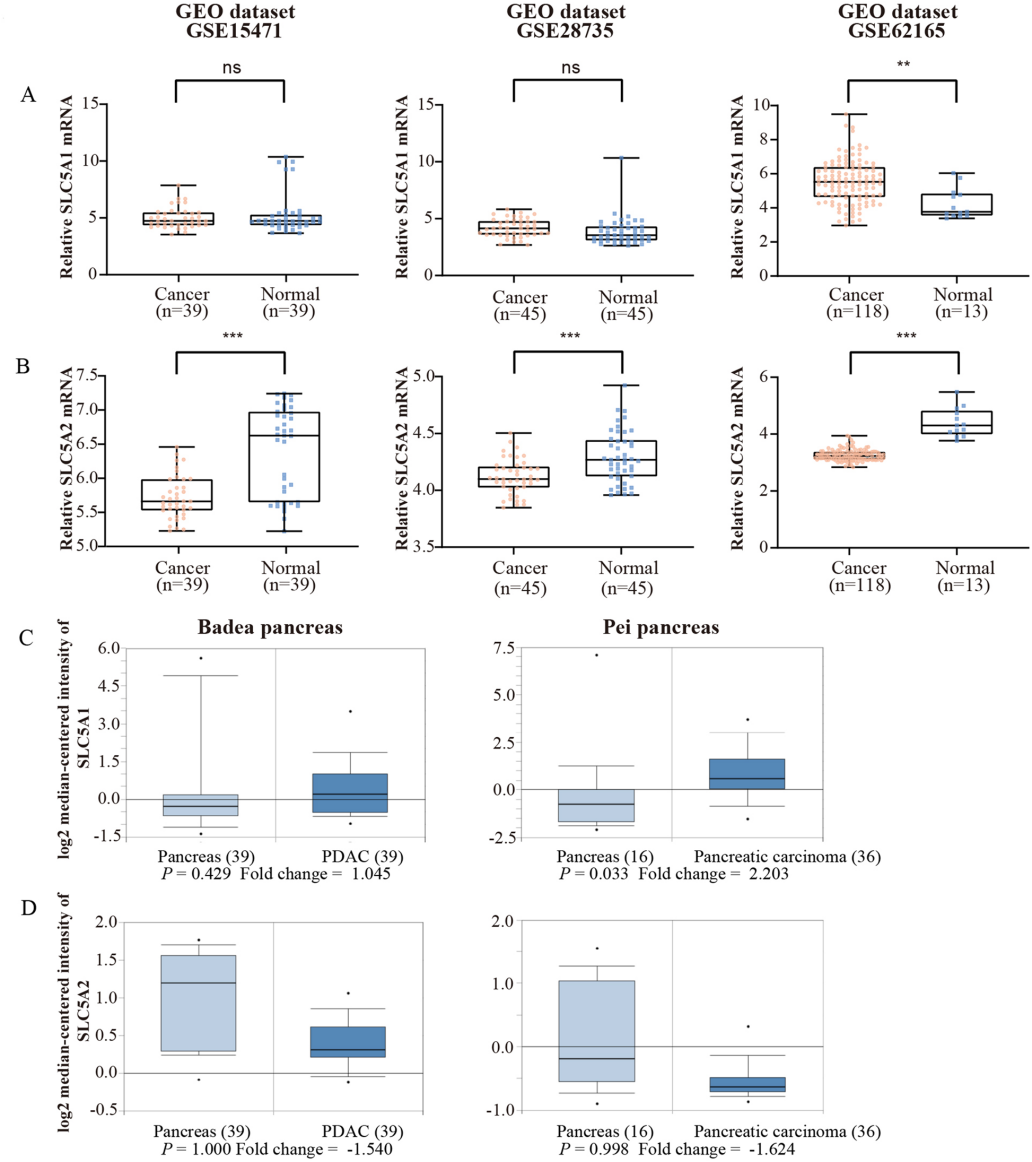
Analysis of SLC5A1 and SLC5A2 RNA expression on the basis of the GEO database (GEO series 15471, 28735 and 62165) and Oncomine database (Badea Pancreas and Pei Pancreas). **A** Statistical analysis of the mRNA level of SLC5A1 between tumour and normal tissues in the GEO database. **B** Statistical analysis of the mRNA level of SLC5A2 between tumour and normal tissues in the GEO database. **C** Statistical analysis of the mRNA level of SLC5A1 between tumour and normal tissues in the Oncomine database. **D** Statistical analysis of the mRNA level of SLC5A2 between tumour and normal tissues in the Oncomine database (^*^ for *P <* 0.05, ^**^ for *P <* 0.01, ^***^ for *P <* 0.001; error bar: minimum to maximum; ns: not significant)

The details of the GEO series included in this analysis are summarized in Table 2. The expression level of SLC5A1 mRNA in tumour tissue was significantly higher than that in normal tissue in GSE62165 (*P* = 0.001, Table 2 and Fig Fig. 2A). The expression level of SLC5A2 mRNA in tumour tissue was significantly lower than that in normal tissue in GSE15471, GSE28735 and GSE62165 (all *P <* 0.001, Table 2 and Fig. 2B). The Oncomine database indicated that SLC5A1 mRNA levels were significantly higher in tumours in the Pei Pancreas dataset (*P =* 0.033) but not in the Badea Pancreas dataset (*P =* 0.429, Fig. 2C). SLC5A2 mRNA levels were lower in tumour tissue in both datasets (not significant, Fig. 2D). *P* value and Fold change value in Fig 2C and D was automatically generated by Oncomine.

**Table 2:** Details of GEO series and results of bioinformatics analysis

Dataset	Gene symbol	Type	Number	Mean	*P*
GSE15471	SLC5A1	PDAC	39	5.101	0.293
		Adjacent	39	5.312	
	SLC5A2	PDAC	39	5.720	< 0.001^*^
		Adjacent	39	6.397	
GSE28735	SLC5A1	PDAC	45	4.169	0.131
		Adjacent	45	3.870	
	SLC5A2	PDAC	45	4.119	< 0.001^*^
		Adjacent	45	4.294	
GSE62165	SLC5A1	PDAC	118	5.532	0.001^*^
		Adjacent	13	4.251	
	SLC5A2	PDAC	118	3.258	< 0.001^*^
		Adjacent	13	4.404	

*GSE* GEO series

^*^Statistical significance

### Association between SGLT-1 expression and prognosis in PDAC patients

#### Association between SGLT-1 expression and prognosis in PDAC patients in all TNM stages

**Fig. 3:**
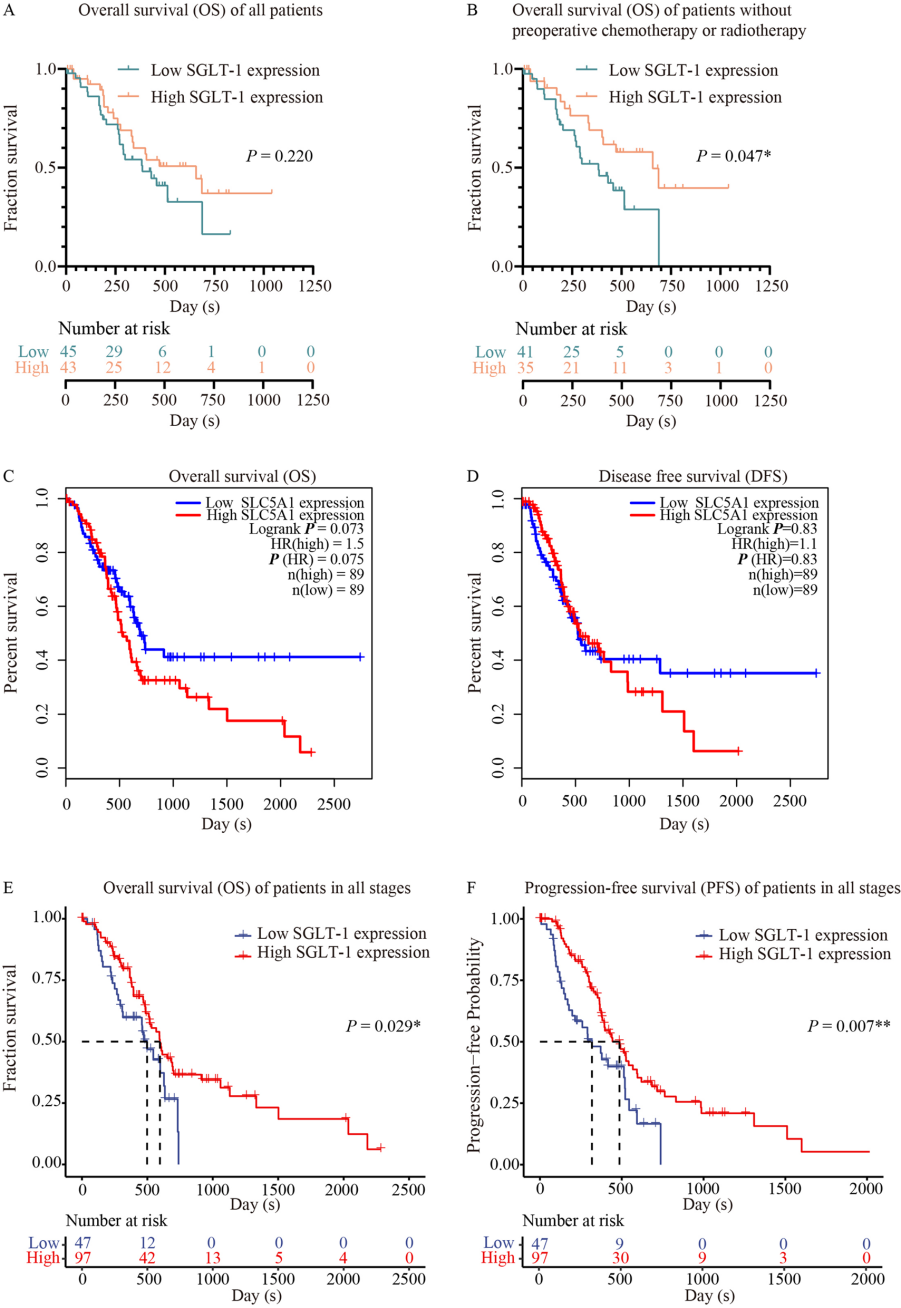
Correlation between SGLT-1 (SLC5A1) and prognosis in PC. **A** and **B** Kaplan–Meier analysis of OS for patients with high vs. low SGLT-1 expression in IHC. **C** Kaplan–Meier analysis of OS for patients with high vs. low SGLT-1 (SLC5A1) mRNA expression using GEPIA. **D** Kaplan–Meier analysis of DFS for patients with high vs. low SGLT-1 (SLC5A1) mRNA expression using GEPIA. **E** Kaplan–Meier analysis of OS for patients with high vs. low SGLT-1 (SLC5A1) mRNA expression based on data downloaded from TCGA. **F** Kaplan–Meier analysis of PFS for patients with high vs. low SGLT-1 (SLC5A1) mRNA expression based on data downloaded from TCGA. (^*^ for *P <* 0.05, ^**^ for *P <* 0.01)

Because positive SGLT-2 expression was not found in PC, we investigated the association between the expression level of SGLT-1 and the prognosis of patients with PC. The correlation between SGLT-1 and patient survival was analysed. Scores ≥6 for SGLT-1 were defined as high expression. The median OS in the whole study cohort was 431 days. Kaplan–Meier survival analysis indicated that the median OS in the low SGLT-1 group was 386 days and that in the high SGLT-1 group was 658 days (*P* = 0.220, Fig. 3A). When patients receiving preoperative chemotherapy or radiotherapy were excluded, the median OS in the cohort was 458 days. The median OS in the low SGLT-1 group was 383 days, and that in the high SGLT-1 group was 658 days (*P* = 0.047, Fig. 3B), indicating that high SGLT-1 expression tended to be significantly associated with longer OS.

Because preoperative therapy could affect the expression level of proteins in tumour cells, IHC of samples from these patients could not reflect the original expression level of protein. Thus, the above results from patients without preoperative chemotherapy or radiotherapy were more persuasive and could better reflect this fact. Therefore, the predictive value of SGLT-1 for the prognosis of patients who underwent surgery directly without preoperative chemotherapy or radiotherapy was confirmed in Cox regression analysis. Through univariate analysis, it was found that TNM stage III was a predictive factor for poor prognosis (HR = 3.080, 95% CI 1.172–8.089, *P* = 0.022, Table 3). Multivariate analysis indicated that TNM stage III (*P* = 0.020) and pathological grade (*P* = 0.034) were independent factors for poor prognosis, while high SGLT-1 expression (HR = 0.419, 95% CI 0.207–0.846, *P* = 0.015) was an independent factor for a better prognosis (Table 3).

**Table 3:** Univariate and multivariate analyses for predictive factors of PDAC survival

Variables	Univariable		Multivariable	
	HR (95% CI)	*P*	HR (95% CI)	*P*
Age (years)		0.176		0.103
< 64	Ref		Ref	
≥ 64	1.578 (0.815–3.058)		–	
Sex		0.993		0.269
Female	Ref		Ref	
Male	0.997 (0.493–2.015)		–	
TNM stage		0.074		0.062
I	Ref		Ref	
II	2.591 (0.830–8.087)	0.101	3.111 (0.979–9.886)	0.054
III	3.080 (1.172–8.089)	0.022^*^	3.145 (1.198–8.260)	0.020^*^
Grade		0.053		0.034^*^
I	Ref		Ref	
II	1.843 (0.883–3.874)	0.104	2.257 (1.044–4.882)	0.039^*^
III	2.091 (0.909–4.811)	0.083	2.881 (1.190–6.977)	0.019^*^
SMV/SMA invasion		0.385		0.747
No	Ref		Ref	
Yes	1.331 (0.699–2.536)		–	
History of diabetes		0.637		0.550
No	Ref		Ref	
Yes	1.209 (0.549–2.666)		–	
SGLT-1		0.051		0.015^*^
Low	Ref		Ref	
High	0.508 (0.257–1.004)		0.419 (0.207–0.846)	

*SMV* Superior mesenteric vein, *SMA* Superior mesenteric artery, *HR* Hazard ratio, *CI* Confidence interval

^*^Statistical significance

After that, to make our findings more comprehensive and persuasive, survival analysis was performed again using the GEPIA database based on the TCGA. However, the mRNA expression level of SGLT-1 (SLC5A1) was not significantly correlated with OS or DFS (*P* = 0.073 and *P* = 0.83, respectively), and SGLT-1 (SLC5A1) was not shown to be an independent risk factor for OS (*P* = 0.075) or DFS (*P* = 0.83) (Fig. 3C and D).

Given that GEPIA database did not provide original dataset thus the histologic type of cohort PAAD (pancreatic adenocarcinoma) chosen here might be of great heterogeneity. And only 89 samples were included in this survival analysis. Thus, survival analysis and univariate and multivariate analysis was performed again based on data directly downloaded from TCGA-PAAD dataset. 144 PDAC patients without preoperative chemotherapy or radiotherapy were finally enrolled in this study, whose characteristics was summarised in Table 4. Kaplan–Meier survival analysis indicated that the median OS in the low SGLT-1 (SLC5A1) group was 498 days and that in the high SGLT-1 (SLC5A1) group was 596 days (*P* = 0.029, Fig. 3E). And median PFS (progression free survival) in the low SGLT-1 (SLC5A1) group was 318 days and that in the high SGLT-1 (SLC5A1) group was 486 days (*P* = 0.007, Fig. 3F). These results indicated that high SGLT-1 expression was significantly associated with longer OS and PFS.

**Table 4:** Clinicopathological characteristics of PDAC patients from TCGA

Features	No. of all patients (%)
Age (years)	144 (100)
<66	73 (50.7)
≥ 66	71 (49.3)
Gender	144 (100)
Female	68 (47.2)
Male	76 (52.8)
T stage	144 (100)
T1	4 (2.8)
T2	15 (10.4)
T3	122 (84.7)
T4	3 (2.1)
N stage	143 (100)
N0	36 (25.2)
N1	107 (74.8)
TNM Stage	144 (100)
I	12 (8.3)
II	126 (87.5)
III	3 (2.1)
IV	3 (2.1)
Grade	144 (100)
G1	20 (13.9)
G2	82 (56.9)
G3	41 (28.5)
G4	1 (0.7)
Surgery type	142 (100)
Distal Pancreatectomy	15 (10.6)
Whipple	115 (81.0)
Total Pancreatectomy	1 (0.7)
Other type	11 (7.7)
Residual tomour	133 (100)
R0	82 (61.7)
R1	46 (34.6)
R2	5 (3.7)
History of diabetes	119 (100)
No	86 (72.3)
Yes	33 (27.7)

#### Association between SGLT-1 expression and prognosis in PDAC patients in TNM stage I - II

**Fig. 4:**
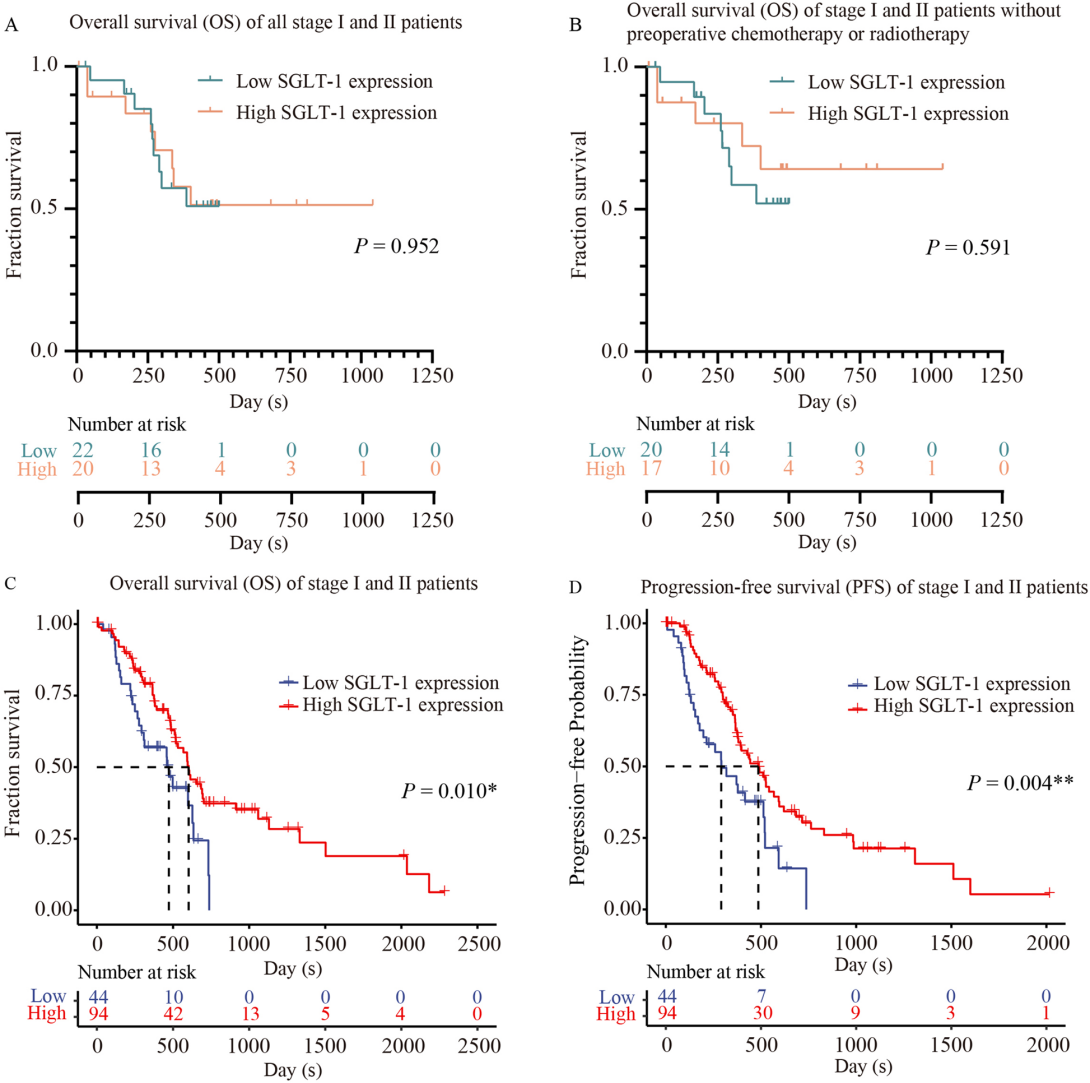
Correlation between SGLT-1 (SLC5A1) and prognosis of PDAC patients in stage I and II. **A** and **B** Kaplan-Meier analysis of OS for stage I and II patients with high vs. low SGLT-1 expression in IHC. **C** Kaplan-Meier analysis of OS for stage I and II patients with high vs. low SGLT-1 (SLC5A1) mRNA expression based on data downloaded from TCGA. **D** Kaplan–Meier analysis of PFS for stage I and II patients with high vs. low SGLT-1 (SLC5A1) mRNA expression based on data downloaded from TCGA. (^*^ for *P <* 0.05, ^**^ for *P <* 0.01)

Then To further study the prognosis predictive value of SGLT-1, Kaplan-Meier survival analysis was performed again in stage I and II patients for whom prognostic indicators are the most needed. Based on data from IHC, it was indicated that the mean OS in stage I and II patients was 650 days, and the mean OS in the low SGLT-1 group was 378 days and 652 days in the high SGLT-1 group (*P* = 0.952, Fig. 4A). When patients receiving preoperative chemotherapy or radiotherapy were excluded, the mean OS in the cohort was 696 days. Mean OS in the low SGLT-1 group was 379 days, and 744 days in the high SGLT-1 group (*P* = 0.591, Fig. 4B), indicating that high expression of SGLT-1 tended to be associated with longer OS but not significantly.

Based on data from TCGA, 6 patients in TNM stage III and IV was excluded and 138 patients in TNM stage I and II were enrolled in the study. Kaplan–Meier survival analysis indicated that the median OS in the low SGLT-1 (SLC5A1) group was 473 days and that in the high SGLT-1 (SLC5A1) group was 603 days (*P* = 0.010, Fig. 4C). And median PFS in the low SGLT-1 (SLC5A1) group was 291 days and that in the high SGLT-1 (SLC5A1) group was 486 days (*P* = 0.004, Fig. 4D). These results indicated that high SGLT-1 expression was significantly associated with longer OS and PFS in stage I and II PDAC patients.

### Potential reason for the specific survival significance of SGLT-1

#### Expression of SGLT-1 was not correlated with pathological degree or TNM stage in PDAC

Pathological degree and TNM stage of PDAC patients have been demonstrated to be risk factors for prognosis based on IHC in Result 4 – Table 3, and high GLUT-1 expression was demonstrated to be associated with poor prognosis of PC in several previous studies, which means that expression of SGLT-1 has opposite survival relevance to these three factors [34–39]. Given the opposite stage/grade-specific distribution of SGLT-2 and GLUT-1 in LDAC stated before, we then analysed the correlation between SGLT-1 and TNM stage, pathological grade and expression of GLUT-1 respectively to explore whether the special opposite survival significance of SGLT-1 was derived from or associated with a similar stage/grade-specific distribution of SGLT-1 in PDAC to SGLT-2 and GLUT-1 in LDAC.

**Fig. 5:**
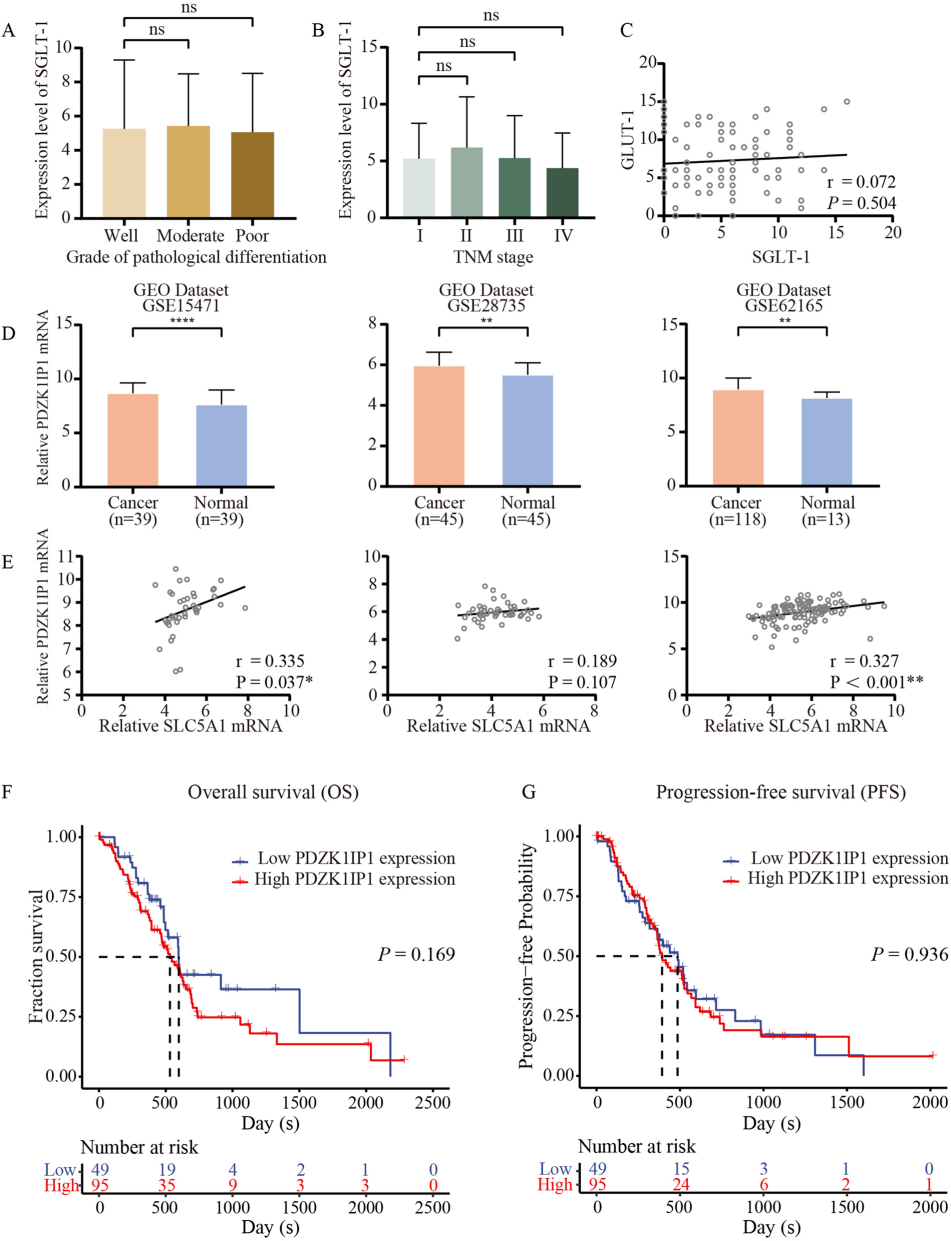
Potential reason for the survival significance of SGLT-1 in PDAC. **A** Expression level of SGLT-1 across different grades of pathological differentiation in IHC. **B** Expression level of SGLT-1 across different TNM stages in IHC. **C** The correlation between the expression level of GLUT-1 and SGLT-1 in IHC. **D** Statistical analysis of the mRNA level of MAP17 (PDZK1IP1) between tumour and normal tissues in pancreatic cancer in the GEO database (GEO series 15471, 28735 and 62165). **E** Relationships between the mRNA levels of SGLT-1 (SLC5A1) and MAP17 (PDZK1IP1) in pancreatic cancer on the basis of the GEO database in the same series. **F** Kaplan–Meier analysis of OS for patients with high vs. low MAP17 (PDZK1IP1) mRNA expression based on data downloaded from TCGA. **G** Kaplan–Meier analysis of PFS for patients with high vs. low MAP17 (PDZK1IP1) mRNA expression based on data downloaded from TCGA. Pearson’s correlations were used to estimate the correlation efficiency and statistical significance (^**^ for *P <* 0.01, ^***^ for *P <* 0.001, ^****^ for *P <* 0.0001, ns: not significant, error bar: standard deviation)

Table 1 shows that no correlation was observed between the expression of SGLT-1 and pathological grade or TNM stage. Thus, one-way ANOVA was performed to further analyse the correlation between SGLT-1 and the two main clinicopathological characteristics. No significant difference was observed in the expression of SGLT-1 between different pathological grades (*P* = 0.953, Fig. 5A) or TNM stages (*P* = 0.569, Fig. 5B). Pearson Correlation analysis indicated that there was no significant correlation between the two glucose transporters in Pearson correlation analysis (r = 0.072, *P* = 0.504, Fig. 5C).

#### MAP17 was overexpressed in PDAC, and the expression level of SGLT-1 was correlated with MAP17

The expression level of MAP17 (PDZK1-interacting protein 1, PDZK1IP1) was assessed in the three expression microarray series. The expression level of MAP17 in pancreatic cancer tissue was significantly higher than that in normal tissue (Fig. 5D, *P* < 0.0001 in GSE15471, *P* = 0.002 in GSE28735, *P* = 0.009 in GSE62165). The expression level of SGLT-1 was significantly correlated with the expression level of MAP17 in GSE15471 (*r* = 0.335, *P* = 0.037) and GSE62165 (*r* = 0.327, *P* <  0.001) (Fig. 5E).

Then survival analysis and cox regression were performed based on the mRNA level of MAP-17 (PDZK1IP1) in TCGA-PAAD cohort to examine whether MAP-17 (PDZK1IP1) had consistent survival relevance with SGLT-1 (SLC5A1). The median OS in the low MAP-17 (PDZK1IP1) group was 598 days and that in the high MAP-17 (PDZK1IP1) group was 532 days (*P* = 0.169, Fig. 5F). And the median PFS in the low MAP-17 (PDZK1IP1) group was 486 days and that in the high MAP-17 (PDZK1IP1) group was 393 days (*P* = 0.936, Fig. 5G).

### Univariate and multivariate analysis of predictive factors for prognosis and disease progression based on TCGA-PAAD cohort

#### Univariate and multivariate analysis in PDAC patients in all TNM stages

Through univariate analysis, it was found that residual tumour status (R1 and R2) was a predictive factor for a shorter OS (HR = 1.765, 95% CI 1.104-2.822, *P* = 0.018), and high SGLT-1 (SLC5A1) expression (HR = 0.593, 95% CI 0.369-0.953, *P* = 0.031) was an independent factor for a longer OS (Table 5). High expression of MAP-17 (PDZK1IP1) could not be an independent predictive factor for OS of PDAC patients in all TNM stages (HR = 1.394, 95% CI 0.867-2.243, *P* = 0.170). Factors with *P* <  0.1 were enrolled in multivariate analysis, which indicated that residual tumour status (R1 and R2) was a predictive factor for a shorter OS (HR = 1.825, 95% CI 1.136-2.931, *P* = 0.013), and high SGLT-1 (SLC5A1) expression (HR = 0.593, 95% CI 0.362-0.970, *P* = 0.038) was an independent factor for a longer OS (Table 5).

**Table 5:** Univariate analysis and multivariate analysis for survival predictive factors of PDAC in TCGA

Variables	Univariable		Multivariable	
	HR (95%CI)	*P*	HR (95%CI)	*P*
Age (≥ 66 vs. < 66)	0.971 (0.629-1.498)	0.893	–	–
Gender (Male vs. Female)	0.852 (0.552-1.316)	0.471	–	–
T stage (T3-T4 vs. T1-T2)	1.165 (0.580-2.339)	0.669	–	–
N stage (N2 vs. N1)	1.376 (0.805-2.349)	0.243	–	–
TNM Stage (II vs. I)	0.962 (0.416-2.223)	0.928	–	–
(III vs. I)	0.489 (0.059-4.074)	0.508	–	–
(IV vs. I)	1.279 (0.255-6.412)	0.765	–	–
Grade (G2 vs. G1)	1.078 (0.540-2.152)	0.831	–	–
(G3 vs. G1)	1.432 (0.691-2.971)	0.334	–	–
(G4 vs. G1)	1.228 (0.156-9.676)	0.845	–	–
Surgery type (Whipple and TP vs. DP and other type)	1.839 (0.960-3.524)	0.066	1.643 (0.813-3.321)	0.167
Residual tumour (R1-R2 vs R0)	1.765 (1.104-2.822)	0.018^*^	1.825 (1.136-2.931)	0.013^*^
History of diabetes (Yes vs. No)	0.981 (0.557-1.728)	0.947	–	–
SGLT-1 (SLC5A1) (High vs. Low)	0.593 (0.369-0.953)	0.031^*^	0.593 (0.362-0.970)	0.038^*^
MAP-17 (PDZK1IP1) (High vs. Low)	1.394 (0.867-2.243)	0.170		

*TP* Total Pancreatectomy, *DP* Distal Pancreatectomy

^*^ Statistical significance

And Cox analysis was performed again to find predictive factors for disease progression. Through univariate analysis, it was found that residual tumour status (R1 and R2) was a predictive factor for disease progression and a shorter PFS (HR = 2.309, 95% CI 1.486-3.587, *P* < 0.001), and high SGLT-1 (SLC5A1) expression (HR = 0.547, 95% CI 0.350-0.855, *P* = 0.008) was an independent factor for a longer PFS (Table 6). High expression of MAP-17 (PDZK1IP1) could not be an independent predictive factor for PFS of PDAC patients in all TNM stages (HR = 1.018, 95% CI 0.663-1.563, *P* = 0.934). Factors with *P* < 0.1 were enrolled in multivariate analysis, which indicated that residual tumour status (R1 and R2) was a predictive factor for disease progression and a shorter PFS (HR = 2.590, 95% CI 1.644-4.080, *P* < 0.001), and high SGLT-1 (SLC5A1) expression (HR = 0.534, 95% CI 0.336-0.848, *P* = 0.007) was an independent factor for a longer PFS (Table 6).

**Table 6:** Univariate analysis and multivariate analysis for disease progression predictive factors of PDAC in TCGA

Variables	Univariable		Multivariable	
	HR (95%CI)	*P*	HR (95%CI)	*P*
Age (≥ 66 vs. < 66)	0.943 (0.623-1.428)	0.782	–	–
Gender (Male vs. Female)	1.042 (0.690-1.573)	0.844	–	–
T stage (T3-T4 vs. T1-T2)	1.297 (0.668-2.519)	0.442	–	–
N stage (N2 vs. N1)	1.327 (0.814-2.163)	0.257	–	–
TNM Stage (II vs. I)	1.181 (0.513-2.723)	0.696	–	–
(III vs. I)	1.169 (0.235-5.826)	0.848	–	–
(IV vs. I)	1.077 (0.216-5.382)	0.927	–	–
Grade (G2 vs. G1)	0.799 (0.432-1.478)	0.474	0.690 (0.349-1.364)	0.286
(G3 vs. G1)	1.253 (0.649-2.417)	0.501	1.182 (0.579-2.414)	0.646
(G4 vs. G1)	7.490 (0.932-60.216)	0.058	4.819 (0.573-40.528)	0.148
Surgery type (Whipple and TP vs. DP and other type)	1.377 (0.784-2.418)	0.265	–	–
Residual tumour (R1-R2 vs R0)	2.309 (1.486-3.587)	< 0.001^*^	2.590 (1.644-4.080)	< 0.001^*^
History of diabetes (Yes vs. No)	0.715 (0.411-1.245)	0.236	–	–
SGLT-1 (SLC5A1) (High vs. Low)	0.547 (0.350-0.855)	0.008^*^	0.534(0.336-0.848)	0.007^*^
MAP-17 (PDZK1IP1) (High vs. Low)	1.018 (0.663-1.563)	0.934		

*TP* Total Pancreatectomy, *DP* Distal Pancreatectomy

^*^ Statistical significance

#### Univariate and multivariate analysis in PDAC patients in TNM stage I - II

Through univariate analysis, it was found that residual tumour status (R1 and R2) was a predictive factor for a shorter OS (HR = 1.683, 95% CI 1.040-2.723, *P* = 0.034), and high SGLT-1 (SLC5A1) expression (HR = 0.533, 95% CI 0.328-0.866, *P* = 0.011) was an independent factor for a longer OS (Table 7). High expression of MAP-17 (PDZK1IP1) could not be an independent predictive factor for OS of PDAC patients in TNM stage I - II (HR = 1.273, 95% CI 0.788-2.055, *P* = 0.324). Factors with *P* < 0.1 were enrolled in multivariate analysis, which indicated that residual tumour status (R1 and R2) was a predictive factor for a shorter OS (HR = 1.753, 95% CI 1.080-2.847, *P* = 0.023), and high SGLT-1 (SLC5A1) expression (HR = 0.526, 95% CI 0.317-0.875, *P* = 0.013) was an independent factor for a longer OS (Table 7).

**Table 7:** Univariate analysis and multivariate analysis for survival predictive factors of PDAC patients at TNM stage I to II in TCGA

Variables	Univariable		Multivariable	
	HR (95%CI)	*P*	HR (95%CI)	*P*
Age (≥ 66 vs. < 66)	1.058 (0.680-1.646)	0.803	–	–
Gender (Male vs. Female)	0.886 (0.569-1.379)	0.591	–	–
T stage (T3-T4 vs. T1-T2)	1.172 (0.582-2.356)	0.657	–	–
N stage (N2 vs. N1)	1.413 (0.804-2.484)	0.230	–	–
TNM Stage (II vs. I)	0.960 (0.415-2.217)	0.923	–	–
Grade (G2 vs. G1)	1.044 (0.521-2.091)	0.903	–	–
(G3 vs. G1)	1.371 (0.658-2.853)	0.400	–	–
(G4 vs. G1)	1.198 (0.152-9.445)	0.864	–	–
Surgery type (Whipple and TP vs. DP and other type)	1.923 (0.974-3.798)	0.060	1.645 (0.782-3.460)	0.189
Residual tumour (R1-R2 vs R0)	1.683 (1.040-2.723)	0.034^*^	1.753 (1.080-2.847)	0.023^*^
History of diabetes (Yes vs. No)	0.984 (0.550-1.759)	0.956	–	–
SGLT-1 (SLC5A1) (High vs. Low)	0.533 (0.328-0.866)	0.011^*^	0.526(0.317-0.875)	0.013^*^
MAP-17 (PDZK1IP1) (High vs. Low)	1.273 (0.788-2.055)	0.324		

*TP* Total Pancreatectomy, *DP* Distal Pancreatectomy

^*^ Statistical significance

And Cox analysis was performed again to find predictive factors for disease progression in stage I and II patients. Through univariate analysis, it was found that residual tumour status (R1 and R2) was a predictive factor for disease progression and a shorter PFS (HR = 2.200, 95% CI 1.399-3.461, *P* = 0.001), and high SGLT-1 (SLC5A1) expression (HR = 0.510, 95% CI 0.322-0.809, *P* = 0.004) was an independent factor for a longer PFS (Table 8). High expression of MAP-17 (PDZK1IP1) could not be an independent predictive factor for PFS of PDAC patients in TNM stage I - II (HR = 0.971, 95% CI 0.627-1.502, *P* = 0.894). Factors with *P* < 0.1 were enrolled in multivariate analysis, which indicated that residual tumour status (R1 and R2) was a predictive factor for disease progression and a shorter PFS (HR = 2.723, 95% CI 1.678-4.419, *P* < 0.001), and high SGLT-1 (SLC5A1) expression (HR = 0.454, 95% CI 0.280-0.737, *P* = 0.001) was an independent factor for a longer PFS (Table 8).

**Table 8:** Univariate analysis and multivariate analysis for progression predictive factors of PDAC patients at TNM stage I to II in TCGA

Variables	Univariable		Multivariable	
	HR (95%CI)	*P*	HR (95%CI)	*P*
Age (≥ 66 vs. < 66)	0.98 (0.641-1.498)	0.926	–	–
Gender (Male vs. Female)	1.106 (0.725-1.687)	0.641	–	–
T stage (T3-T4 vs. T1-T2)	1.300 (0.668-2.528)	0.440	–	–
N stage (N2 vs. N1)	1.283 (0.770-2.137)	0.338	–	–
TNM Stage (II vs. I)	1.179 (0.512-2.719)	0.699	–	–
Grade (G2 vs. G1)	0.878 (0.464-1.662)	0.690	0.765 (0.375-1.558)	0.460
(G3 vs. G1)	1.372 (0.696-2.706)	0.361	1.492 (0.712-3.127)	0.289
(G4 vs. G1)	8.307 (1.023-67.451)	0.048^*^	5.827 (0.683-49.722)	0.107
Surgery type (Whipple and TP vs. DP and other type)	1.331 (0.743-2.385)	0.336	–	–
Residual tumour (R1-R2 vs R0)	2.200 (1.399-3.461)	0.001^*^	2.723 (1.678-4.419)	< 0.001^*^
History of diabetes (Yes vs. No)	0.647 (0.36-1.161)	0.144	–	–
SGLT-1 (SLC5A1) (High vs. Low)	0.510 (0.322-0.809)	0.004^*^	0.454 (0.280-0.737)	0.001^*^
MAP-17 (PDZK1IP1) (High vs. Low)	0.971 (0.627-1.502)	0.894		

*TP* Total Pancreatectomy, *DP* Distal Pancreatectomy

^*^ Statistical significance

## Discussion

Different from GLUT-1, SGLTs are another kind of glucose transporter harnessing a gradient of sodium ions across the plasma membrane to drive glucose and other nutrients into cells [40]. The most studied family members are SGLT-1 and SGLT-2, which are functionally involved in glucose transport in the intestine and kidneys as well as in specialized regions of the brain [7]. Scafoglio et al. identified the functional expression of SGLTs in prostate cancer and pancreatic cancer in 2015 and in LADC in 2018 [8, 9]. SGLT-1 showed predominant nuclear staining in malignant duct cells in PDAC and was specifically stained on the apical surfaces of intra- and interlobular ducts in adjacent normal pancreas tissue, while cytoplasmic staining of SGLT-1 was only observed in two out of six PDAC samples [8]. However, in our study, positive SGLT-1 staining was found predominantly in the cytoplasm and some membranes, while little staining was observed in the nucleus of malignant duct cells of PDAC and barely in adjacent normal tissue (mean score of tumour vs. normal tissue 5.273 vs. 0.760, *P* < 0.0001, Fig. 1D), consistent with the conclusion of the study by Casneuf et al. [41]. In addition, specific SGLT-1 staining was observed in islet cells in our study (Fig. 1B and C). Furthermore, supported by bioinformatics analysis, the level of SLC5A1 in PDAC tumour tissue was higher than that in normal tissue in two of the three GEO series (Fig. 2A) and in the Pei Pancreas dataset from the Oncomine database (Fig. 2C).

In our opinion, since SGLT-1 is a membrane-expressed protein actively transporting glucose and glucose metabolism takes place in the cytoplasm, it is difficult to elaborate the biological functions of SGLT-1 found in the nucleus of PDAC cells by Scafoglio et al. Additionally, there were few studies by other laboratories reporting the translocation of SGLT-1 to nuclei. Thus, the accuracy of the results regarding the subcellular localization of SGLT-1 in the study by Scafoglio et al. might need more convincing experiments to prove and explain the finding.

Since there was no retrospective study on the expression of SGLT-2 in PC before, thus, to study the expression of SGLT-2 and confirm the specificity of the IHC staining, IHC was performed twice on serial slices with antibodies from two different companies respectively, and little positive staining was observed in our 76 PDAC samples, completely opposite of the results of the robust staining of SGLT-2 in their study [9]. The only specific strong SGLT-2 staining was observed in islet cells in our study (Fig. 1E, F, G, Fig. S1A and B), similar to SGLT-1. This was supported by the finding that the expression of SGLT-1 and SGLT-2 in pancreatic alpha cells was identified at the mRNA and protein levels by Bonner et al. [40, 42]. To further verify our results regarding SGLT-2, bioinformatics analysis was performed. In the GEO dataset, the expression level of SLC5A2 mRNA in tumour tissue was significantly lower than that in normal tissue in the three series (all *P* < 0.001, Fig. 2B and Table 2). In the Oncomine database, SLC5A2 mRNA levels were lower in tumour tissue in both datasets (not significant, Fig. 2D).

Given the expression of SGLT-2 in pancreatic islet alpha cells evidenced in the study of Bonner et al. and ours, we suppose that the higher mRNA level of SLC5A2 in normal pancreatic tissues might be due to the robust functional expression of SLC5A2 on islet alpha cells. In the process of RNA extraction, islet alpha cells would be counted as normal tissue and contribute to the expression level of genes in mRNA abundance detection, which could appear more significant in statistical analysis in larger samples. On the other hand, we only counted the staining on normal pancreatic duct cells but not islet cells in normal tissues as the score of SGLT-2 in IHC. This led to the expression of SGLT-2 in normal tissues being lower than that in tumour tissues; thus, it would be the reason why the SGLT-2 expression levels in tumour and normal tissues in IHC (Fig. 1H) were opposite to the results from the bioinformatics analysis (Fig. 2B and D). Additionally, the discrepancy between the two bars in Fig. 1H or Fig. S1C was neither representative nor meaningful because positive SGLT-2 staining was obviously hard to find in both tumoral and normal pancreatic duct cells.

After that, we performed a Kaplan–Meier survival analysis of all PDAC patients according to the expression of SGLT-1. Contrary to GLUT-1, it was demonstrated that lower SGLT-1 expression was associated with shorter OS, and multivariate analysis indicated that SGLT-1 was an independent prognostic factor for patients with PDAC who directly underwent surgery (Table 3). The reason why a significant difference was not found in the cohort of all patients, including those with neoadjuvant therapy, may be that preoperative chemotherapy or radiotherapy could inhibit tumour progression by affecting the biological function of tumour cells, a process in which the original expression level of protein could be changed. Thus, the expression level of glucose transporters shown by IHC in tumours affected by preoperative therapy could not reflect the actual and original expression level in these proteins. Alternatively, the inconsistency in this aspect in medical history could affect the accuracy of statistics, making it difficult to reflect the real information behind the data. Therefore, a significant difference was not found in the survival analysis of all patients, including those receiving preoperative therapy. This intriguing result from survival analysis was consistent with the conclusion of the study by Casneuf et al. that PDAC patients with lower SGLT-1 expression have shorter DFS and OS [41].

Then, Kaplan–Meier analysis and Cox regression were performed again using the GEPIA database based on the mRNA expression level of SGLT-1 (SLC5A1) in the TCGA database. The mRNA level of SGLT-1 (SLC5A1) was not correlated with the OS and DFS of patients with PDAC, and the survival curves of patients with different levels of SGLT-1 (SLC5A1) mRNA intersected, indicating that the prognostic significance of SGLT-1 (SLC5A1) was not as simple and clear as that of GLUT-1 (SLC2A1) in patients with PDAC.

Because GEPIA is only an online analysis tools based on data from TCGA, and it did not provide concrete sample selection process and raw clinical data including important clinicopathological information such as pathological type and neoadjuvant therapy history, etc., this could affect the uniformity of the 89 samples and the effectiveness of conclusions. Therefore, the direct results it provided need to be further studied and verified. Thus, survival analysis and univariate and multivariate analysis was performed again based on data directly downloaded from TCGA-PAAD dataset, from which 144 PDAC patients without preoperative chemotherapy or radiotherapy were enrolled in the study finally. High expression of SGLT-1 (SLC5A1) was demonstrated again to be associated with better prognosis of PDAC patients, no matter in all stage patients or stage I and II patients, for whom prognostic indicators are the most needed. The survival analysis based on the expression level in IHC in stage I and II patients showed that though the mean OS in patients with relatively higher expression of SGLT-1 was longer than that in patients with relatively lower expression of SGLT-1, the difference did not reach statistical significance, which might be owing to the small sample size.

Next, we explored the reason behind the special survival significance of SGLT-1. Previously, Scafoglio et al. delineated the association between GLUT-1 and SGLT-2 in LADC. SGLT-2 is predominantly expressed in early-stage, well-differentiated LADC, and as it progresses to advanced and poorly differentiated cancer, it upregulates GLUT-1 as the dominant transporter [9]. Thus, there might be a similar trend of SGLT-1 in PDAC, leading to its special survival significance. However, regrettably, no significant difference in the expression level of SGLT-1 between different pathological grades or TNM stages was found by either Casneuf et al. or us (Table 1 and Fig. 5A and B), and no correlation between the expression of GLUT-1 and SGLT-1 was found by Pearson correlation analysis (Fig. 5C) [41]. Thus, there might not be a similar trend of SGLT-1 to what was found in LADC, and the special survival significance did not result from the hypothesized different distribution of SGLT-1 across pathological grades and TNM stages.

ROS are beneficially involved in many signalling pathways that control development and maintain cellular homeostasis. Under physiological conditions, a tightly regulated redox balance protects cells from injurious ROS activity, and if the balance is altered, it promotes various pathological conditions, including cancer [16]. MAP17 is overexpressed in a variety of cancer types and enhances the tomourigenic phenotype by increasing intracellular ROS [12, 19]. Notably, it has been reported that the ROS increase induced by MAP17 is SGLT-1-dependent, and inhibition of SGLT-1 could inhibit MAP17-induced ROS increases and proliferation [12, 16, 17]. Other studies on cardiomyocyte death induced by oxidative stress reported that glucose transport through SGLT1 is responsible for NADPH oxidase activation and subsequent increased ROS production in cardiomyocytes under hyperglycaemic conditions, and this process was not associated with GLUT-1 [43]. Knockdown of SGLT-1 in cardiomyocytes could reduce the ROS generation and programmed cell death induced by high glucose conditions [44]. In addition, there is a redox balance in tumour cells. Current “ROS threshold” theories suggest that along with increases in ROS, cell responses change from proliferation to balance and then to cell death after ROS surpass a certain level [45]. Mild-to-moderate levels of ROS are associated with the activation of protomourigenic survival and growth pathways, while excessive concentrations of ROS can lead to the induction of cell cycle arrest and cell death [20, 25, 26]. In accordance with this, it has been reported that higher expression of MAP17 and SGLT-1 was correlated with better prognosis in laryngeal and cervical cancers [10, 11].

Based on the above studies, we evaluated the expression level of MAP17 in pancreatic cancer and found that MAP17 was overexpressed in pancreatic cancer and that its expression level was significantly correlated with SGLT-1. This finding indicates that SGLT-1 might participate in the production of ROS induced by MAP17 in pancreatic cancer as they have been demonstrated in other tumour types. Further accumulation of ROS associated with higher levels of SGLT-1 could raise oxidative stress, create a potentially toxic cellular environment and thus initiate the cell death program. This might be the reason why a higher level of SGLT-1 was observed to be correlated with longer patient survival. Additionally, intermittent high glucose conditions were reported to be more harmful to cardiomyocytes by SGLT-1-induced ROS generation and pyroptosis than constant high glucose [44]. Given the glycaemic variability of patients with pancreatic cancer, pancreatic cancer cells might be more vulnerable to SGLT-1-induced oxidative stress. But when we examined the survival relevance of MAP17 (PDZK1IP1) based on data from TCGA, it was shown that no significant prognosis difference between groups with different MAP-17 (PDZK1IP1) level was observed, and expression of MAP-17 (PDZK1IP1) could not be an independent predictor of patient survival. This means that more in-depth researches were needed to study the survival relevance of MAP-17, explore the function of SGLT-1 during the biological role of MAP17, and explain the special prognosis significance of SGLT-1.

The potential options for therapies targeting redox metabolism in pancreatic cancer include preventing PDAC development and relapse via antioxidants or increasing intracellular ROS levels to make pancreatic cancer cells more vulnerable to oxidative stress-induced cell death. Thus, if our conjecture about the correlation about SGLT-1 and MAP17 could be confirmed in future research, pancreatic cancer patients with high MAP17 and SGLT-1 expression might benefit more from therapies that increase oxidative stress, such as cisplatin and radiotherapy. However, together with the therapeutic opportunities provided by SGLT-1-dependent oxidative stress, new challenges arise. Some studies have shown that the clinical application of antioxidants is associated with an increased incidence rate of cancer, probably because of the deprivation of toxic effects on cancer cells mediated by ROS. Given that many pancreatic cancer patients suffer from diabetes, attention should be given to defining a threshold for hypoglycaemic drugs including SGLT inhibitors to decrease the protumorigenic effect through the inhibition of SGLT-1-induced ROS generation without affecting the toxic effect of oxidative stress on cancer cells.

Taken together, these results demonstrated that SGLT-1, from a different family from GLUT-1, is critical for the glucose uptake of PDAC. Its overexpression has the potential to be a biomarker for the prognosis of PDAC. In addition, SGLT-1 might be a marker for identifying patients more likely to benefit from treatments boosting oxidative stress. In particular, SGLT-2 was first found by Scafoglio et al. to be overexpressed in PDAC, and their article became a milestone in the study of the glucose uptake of PDAC and the role of SGLTs in PDAC. Now we proved that SGLT-1 and MAP17, but not SGLT-2, were overexpressed in PDAC, providing a new reference to this field. And the mechanism of distinctive survival relevance of SGLT-1 and prognostic significance of MAP17 need further research to explore.

Prospectively, the discovery of SGLT-1 expression in PDAC and other early-stage tumours and its unique correlation with survival could shed light on the important diagnostic and therapeutic value of SGLT-1 in PDAC. The overexpression of SGLT-1 in PDAC reported in our study was consistent with the accumulation of the nonmetabolized SGLT-specific tracer Me4FDG in mouse models of pancreatic cancer [8]. Therefore, it is anticipated that the measurement of SGLT-mediated glucose utilization with the tracer Me4FDG in PET might be valuable in the early diagnosis and staging of PDAC. Gliflozins, specific SGLT inhibitors approved by the U.S. Food and Drug Administration (FDA) for the treatment of diabetes, might play a role in the field of tumour treatment by inhibiting the glucose uptake of tumour cells or SGLT-1-dependent ROS generation.

This study has some limitations. First, IHC could only partly reflect the protein expression profile of the whole tumour because of the randomness of the expression level of proteins on each slide derived from only one section in the tumour tissue, and its efficacy would be compromised by subjective factors from the reader in semiquantitative analysis. Second, the patient loss to follow-up rate was somewhat high, leading to a decrease in the effectiveness of survival analysis. Third, the studies of the mechanism of the specific survival significance of SGLT-1 and its role in MAP17-dependent ROS generation in PC were just preliminary explorations, and the findings need to be confirmed with more in-depth research.

## Conclusion

In conclusion, SGLT-1 but not SGLT-2 was overexpressed in PC tumour cells, and its overexpression was a predictor for a better prognosis for PDAC patients. In addition, residual tumour status (R1 and R2) was an risk factor for poor prognosis and disease progression.

## Supplementary Information

**Additional file 1: Figure S1.** IHC analysis of the expression SGLT-2 in PDAC tumour tissue and adjacent normal tissue with different primary antibodies. A. Represent image of expression of SGLT-2 in PDAC using Novus Biologicals NBP1–92384. B. Represent image of expression of SGLT-2 in PDAC using Abcam 85,626. C. The statistical comparison of expression of SGLT-2 between pancreatic cancer and normal pancreatic ducts and acinar cells using Abcam ab85626 antibody in IHC. (Red arrow, tumour cell; blue arrow, islet cells; Sample 1: same sample in Fig. 1F; error bar: standard deviation; ns: not significant).

## Data Availability

The datasets used and/or analysed during the current study are available from the corresponding author on reasonable request.
