# Supplementary figures and images for: The expression and survival significance of sodium glucose transporters in pancreatic cancer
Source: BMC Cancer. 2022 Jan 28;22:116. doi: 10.1186/s12885-021-09060-4 (PMC8796473; doi:10.1186/s12885-021-09060-4)

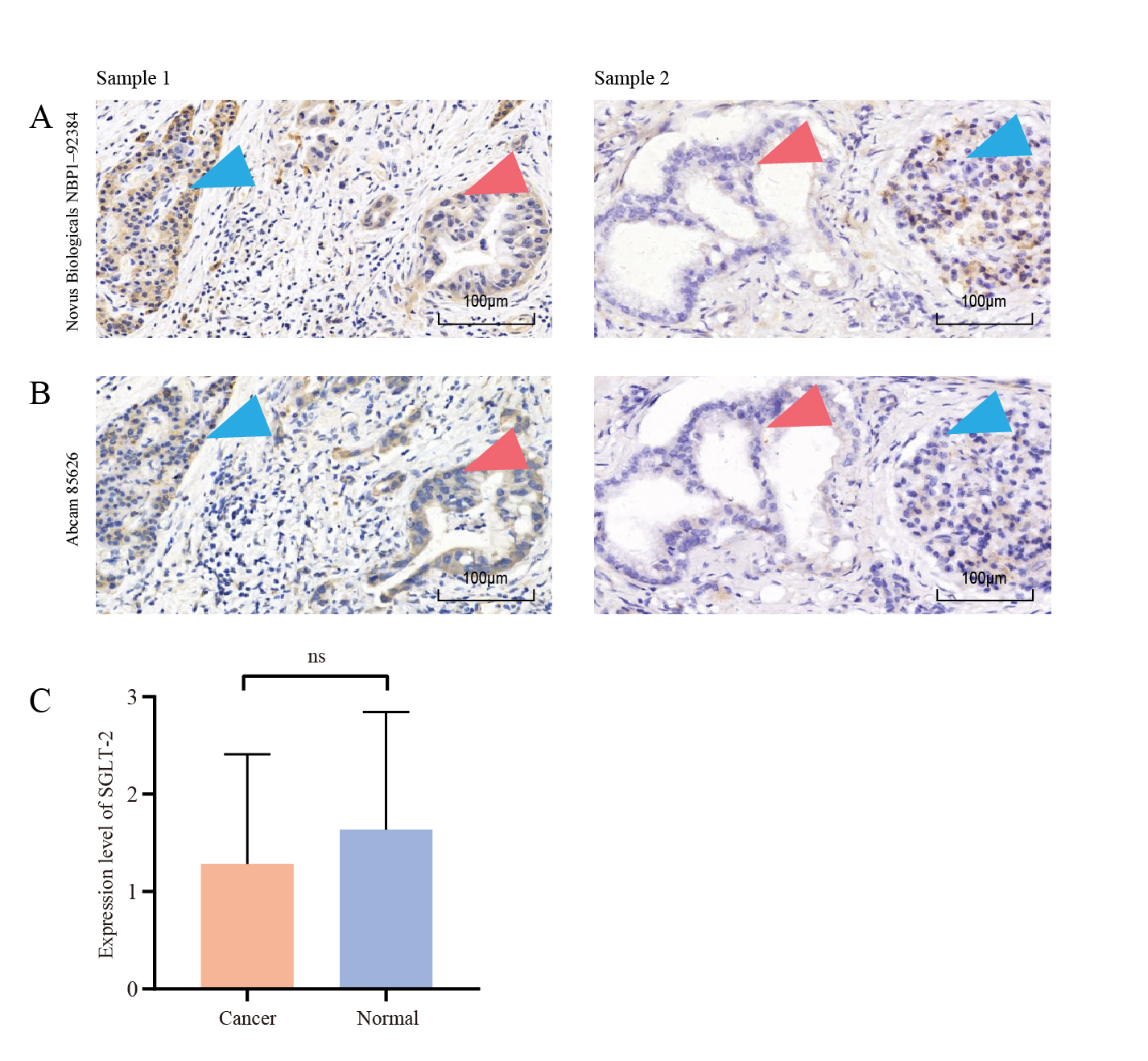

Supplement: Supplementary file 1 — Additional file 1: Figure S1. IHC analysis of the expression SGLT-2 in PDAC tumour tissue and adjacent normal tissue with different primary antibodies. A. Represent image of expression of SGLT-2 in PDAC using Novus Biologicals NBP1–92384. B. Represent image of expression of SGLT-2 in PDAC using Abcam 85,626. C. The statistical comparison of expression of SGLT-2 between pancreatic cancer and normal pancreatic ducts and acinar cells using Abcam ab85626 antibody in IHC. (Red arrow, tumour cell; blue arrow, islet cells; Sample 1: same sample in Fig. 1F; error bar: standard deviation; ns: not significant). [file 12885_2021_9060_MOESM1_ESM.jpg]
